# Initial development and structure of biofilms on microbial fuel cell anodes

**DOI:** 10.1186/1471-2180-10-98

**Published:** 2010-04-01

**Authors:** Suzanne T Read, Paritam Dutta, Phillip L Bond, Jürg Keller, Korneel Rabaey

**Affiliations:** 1 Advanced Water Management Centre, Gehrmann Building, The University of Queensland, Brisbane QLD 4072, Australia

## Abstract

**Background:**

Microbial fuel cells (MFCs) rely on electrochemically active bacteria to capture the chemical energy contained in organics and convert it to electrical energy. Bacteria develop biofilms on the MFC electrodes, allowing considerable conversion capacity and opportunities for extracellular electron transfer (EET). The present knowledge on EET is centred around two Gram-negative models, i.e. *Shewanella *and *Geobacter *species, as it is believed that Gram-positives cannot perform EET by themselves as the Gram-negatives can. To understand how bacteria form biofilms within MFCs and how their development, structure and viability affects electron transfer, we performed pure and co-culture experiments.

**Results:**

Biofilm viability was maintained highest nearer the anode during closed circuit operation (current flowing), in contrast to when the anode was in open circuit (soluble electron acceptor) where viability was highest on top of the biofilm, furthest from the anode. Closed circuit anode *Pseudomonas aeruginosa *biofilms were considerably thinner compared to the open circuit anode (30 ± 3 μm and 42 ± 3 μm respectively), which is likely due to the higher energetic gain of soluble electron acceptors used. The two Gram-positive bacteria used only provided a fraction of current produced by the Gram-negative organisms. Power output of co-cultures Gram-positive *Enterococcus faecium *and either Gram-negative organisms, increased by 30-70% relative to the single cultures. Over time the co-culture biofilms segregated, in particular, *Pseudomonas aeruginosa *creating towers piercing through a thin, uniform layer of *Enterococcus faecium. P. aeruginosa *and *E. faecium *together generated a current of 1.8 ± 0.4 mA while alone they produced 0.9 ± 0.01 and 0.2 ± 0.05 mA respectively.

**Conclusion:**

We postulate that this segregation may be an essential difference in strategy for electron transfer and substrate capture between the Gram-negative and the Gram-positive bacteria used here.

## Background

Microbial fuel cells (MFCs) use bacteria as catalysts to oxidise organic and inorganic matter and generate electrical current. The most widespread proposed use of MFCs, and now the broader term Bioelectrochemical Systems (BESs) [1,2], is for electricity generation during wastewater treatment [3-5]. Irrespective of the goal, the cornerstone of BESs is the capacity of microorganisms to perform or participate in extracellular electron transfer (EET). In this process, microorganisms effectively pump electrons outside the cell, using direct or indirect mechanisms, towards the electron acceptor, i.e. the anode, which is insoluble and exterior to the cell. They also provide us with a platform to perform more fundamental research such as that presented in this paper.

Direct EET occurs via electron flow through outer membrane proteins [6] or potentially through electrically conductive bacterial appendages such as nanowires [7,8] that make physical contact with the anode or other bacteria in the vicinity. Indirect EET involves exogenous (e.g. humics) [9] or endogenous (e.g. phenazines) [10,11] soluble molecules (called mediators or redox shuttles) that act to shuttle electrons through the extracellular aqueous matrix from the cells to the anode [10]. Although there is some evidence that increased current production in Gram-positive bacteria in an MFC is achieved through redox shuttles [12-14], other information pertaining to their role in EET is limited [10,14,15]. Generally, Gram-positive bacteria on their own make limited current in comparison to the Gram-negative [16]. In most cases, the bacteria in the BES grow onto the electrode forming a biofilm, however, to understand the EET process fully we must also investigate the role of the biofilm.

A biofilm is an extracellular polymeric substance (EPS) encased, surface adhering microbial community [17]. Conventional theory categorizes biofilm structure around three basic stages of development, initial attachment, maturation and detachment [17]. The EPS physically immobilize the bacteria while at the same time provide them opportunity for cell to cell contact and communication. Moreover, electron transfer is constrained by the distance over which electrons need to travel to the electron acceptor and therefore, having a greater understanding of biofilm structure and development in BESs may provide us with more of an insight in this area.

Therefore this study aimed (i) to investigate the viability, structure and current production of Gram-positive and -negative pure culture biofilms when growing on a closed circuit (current flowing) and open circuit (soluble electron acceptor provided) anode (ii) to investigate whether bacteria in co-culture generate different levels of current than pure cultures and (iii) to investigate biofilm structure and development between pure and co-cultures on the anode. For this, we used bacteria which had been isolated or used earlier in MFCs: 3 Gram-negatives (G-) *Pseudomonas aeruginosa *PAO1 (*P. aeruginosa*) [18], *Geobacter sulfurreducens *(*G. sulfurreducens*) [8], *Shewanella oneidensis *(*S. oneidensis*) MR-1 [19], and 2 Gram-positive (G+) organisms, *Clostridium acetobutylicum *(*C. acetobutylicum*) [14] and *Enterococcus faecium *(*E. faecium*) [18].

## Results

### Viability of pure culture anode biofilms

**Figure 1:**
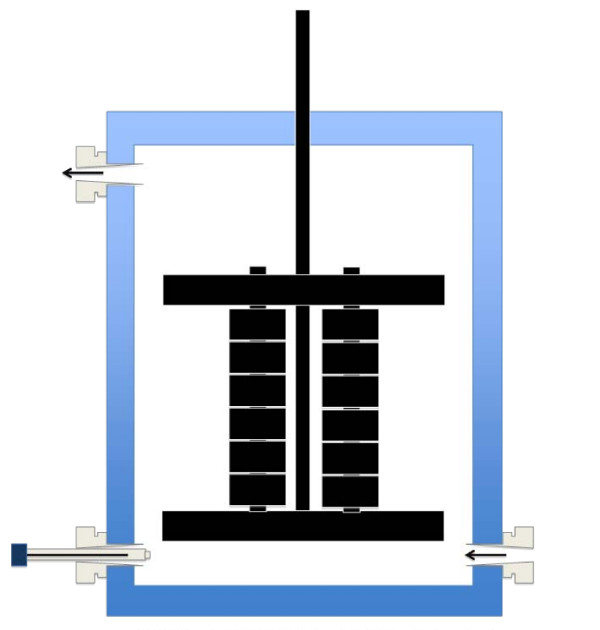
**Schematic of Microbial Fuel cell anode electrode used in all experiments**.

**Figure 2:**
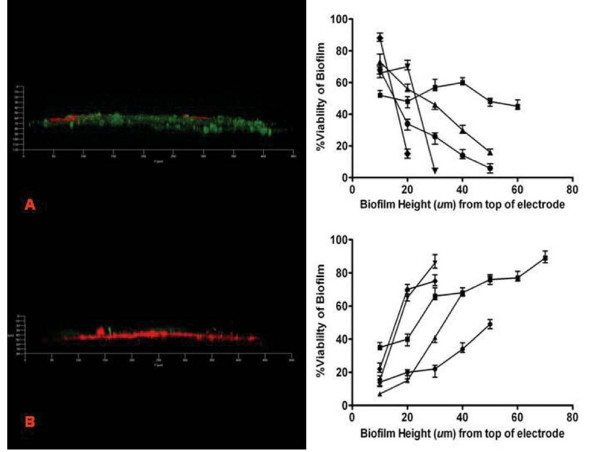
**Graphs showing the relationship between % viability of closed circuit (A) and open circuit (B) MFC experiments**. Biofilm viability increases closer to the anode when the electrode is active. Adjacent CLSM images (20 ×) are both 72 hour side-views of *S. oneidensis *biofilms from batch experiment detected using the Live/Dead (baclight) stain. Circle: *G. sulfurreducens*, Square: *P. aeruginosa*, Upright triangle: *S. oneidensis*, Upsidedown triangle: *E. faecium*and Diamond: *C. acetobutylicum*

Using the five pure cultures, closed circuit (in the presence of anode to cathode current) and open circuit (no current, fumarate and nitrate present) batch experiments were run for three days each in an MFC (Figure 1). During the closed circuit experiments, Live/Dead staining of the biofilm anode blocks indicated that for all species investigated the viability was higher adjacent to the electrode relative to the top of the biofilm. The viability gradually decreased further away from the anode. Additional file 1 demonstrates the higher magnification (63 ×) highlight the staining of the cells and not the matrix which can occur sometimes when using the LIVE/Dead stain. As shown in Figure 2, the viability of *P. aeruginosa *was 44 ± 4% and 76 ± 6% at the top and the bottom of the biofilm respectively (close to anode). In contrast, the open circuit experiments showed greater viability on top of the biofilm, further away from the electrode, while more non-viable areas were detected closer to the electrode. For example, when *P. aeruginosa *was using a soluble electron acceptor the viabilities were 89.3 ± 2.5% and 23.5 ± 3.8% top and bottom respectively (Figure 2B).

### Development and current generation of pure and co-culture anode biofilms

During the pure culture closed circuit experiments the heights of the biofilms were less than that of the open circuit experiments (Table 1). For example, the biofilm height of *P. aeruginosa *was 30 ± 3 μm for the closed circuit experiment and 42 ± 3 μm for the open circuit experiment, as calculated with COMSTAT. All G- cultures developed an ample coverage of the electrode within the three ay period both in closed and open circuit. For example, the *S. oneidensis *biofilm formed large towers of 40 μm high and up to ~50 μm in diameter while the G+ species developed smaller microcolonies with the odd tower up to 20 μm high and 10-20 μm in diameter (during closed circuit). The latter was also reflected in the higher roughness coefficient between the G- and G+ biofilms indicating that during batch mode the G+ are flatter and more uniform than the G- (Table 2). During these pure culture batch experiments G+ species delivered low current throughout while the G- produced a much higher current as shown in Table 1.

**Table 1:** Comparison of current generation and biofilm heights in pure and co-cultures.

	Imax (mA)		Maximum Biofilm thickness (μm, batch)-COMSTAT	
	Continuous	Batch	Closed circuit anode	Open circuit anode
Pure culture experiments				
*Geobacter sulfurreducens*	1.1 ± 0.06	1.0 ± 0.05	25 ± 6	49 ± 5
*Pseudomonas aeruginosa*	0.5 ± 0.01	0.9 ± 0.01	30 ± 3	42 ± 3
*Shewanella oneidensis*	1.3 ± 0.05	1.0 ± 0.15	26 ± 2	41 ± 3
*Clostridium acetobutylicum*	0.13 ± 0.006	0.1 ± 0.03	14 ± 6	24 ± 6
*Enterococcus faecium*	0.1 ± 0.05	0.2 ± 0.05	18 ± 3	23 ± 4
**Co-cultures with *Enterococcus faecium***				

*Geobacter sulfurreducens*	1.9 ± 0.03	-	50 ± 7	-
*Pseudomonas aeruginosa*	1.8 ± 0.04	-	40 ± 4	-
*Shewanella oneidensis*	2.0 ± 0.06	-	39 ± 7	-
**Co-cultures with *Clostridium acetobutylicum***				

*Geobacter sulfurreducens*	0.1 ± 0.03	-	7 ± 3	-
*Pseudomonas aeruginosa*	0.3 ± 0.05	-	8 ± 2	-
*Shewanella oneidensis*	0.2 ± 0.06	-	5 ± 1	-

**Table 2:** Roughness coefficients of biofilms determine by COMSTAT.

	Roughness Coefficient -Batch		Roughness Coefficient -continuous
	Closed circuit anode	Open circuit anode	
Pure culture experiments			
*Geobacter sulfurreducens*	1.8 ± 0.3	1.0 ± 0.4	1.8 ± 0.2
*Pseudomonas aeruginosa*	1.8 ± 0.5	1.1 ± 0.2	1.9 ± 0.1
*Shewanella oneidensis*	1.7 ± 0.2	0.9 ± 0.3	1.9 ± 0.3
*Clostridium acetobutylicum*	1.5 ± 0.3	1.2 ± 0.3	1.7 ± 0.2
*Enterococcus faecium*	1.4 ± 0.2	1.2 ± 0.2	1.9 ± 0.3
**Co-culture experiments with** *Enterococcus faecium*			

*Geobacter sulfurreducens*	-	-	0.9 ± 0.2
*Pseudomonas aeruginosa*	-	-	0.8 ± 0.1
*Shewanella oneidensis*	-	-	0.7 ± 0.1

**Figure 3:**
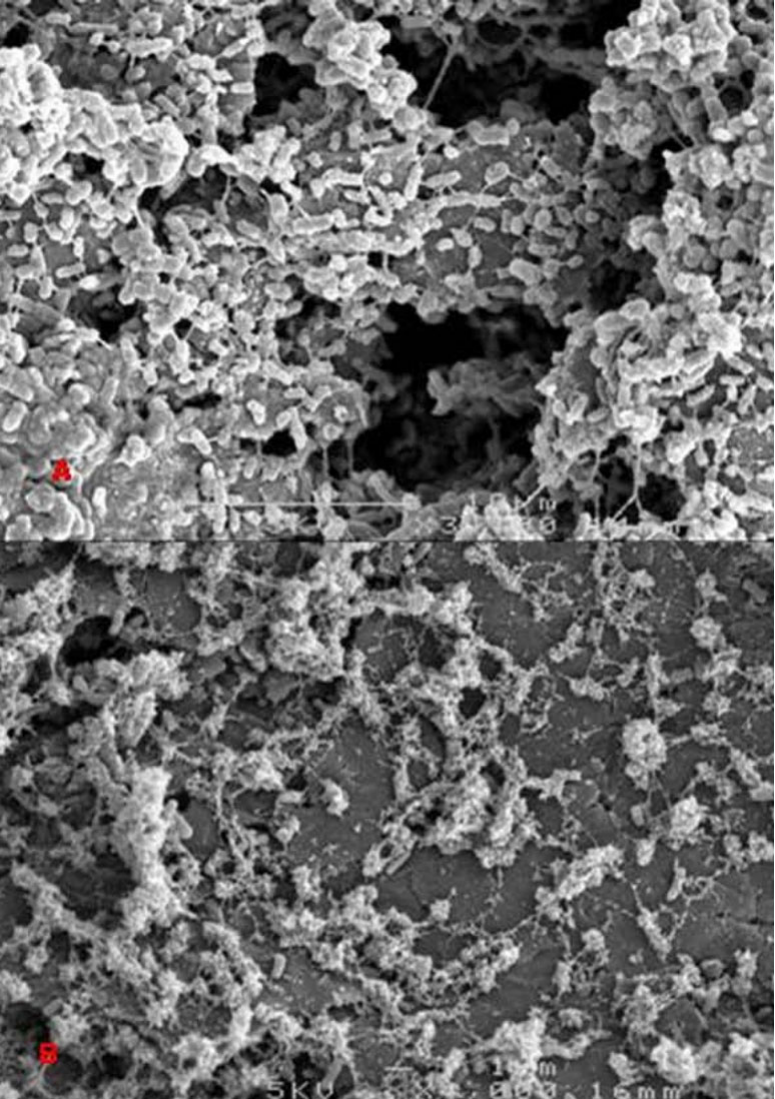
**SEM images of *P. aeruginosa *biofilms at A. 72 hours (3000 ×) and B. 144 hours (3000 ×) during continuous mode**.

During the pure culture continuous experiments, *G. sulfurreducens *and *S. oneidensis *initially showed very similar development, although slower than *P. aeruginosa*, with small towers averaging a height of 8 μm and diameters between 10-20 μm. Moreover, the biofilms became less dense with higher towers developing while prolonged biofilm development revealed less coverage of the electrode giving way to the formation of channels and loss of biofilm mass, similar to that observed in the *P. aeruginosa *biofilm (Figure 3). Additionally, a few towers reaching 50 μm in height were observed in the *G. sulfurreducens *biofilm while the *S. oneidensis *biofilm revealed an occasional tower structure up to 45 μm dispersed throughout the biofilm. These results also correlated with the high level of roughness coefficient measurement from COMSTAT (Table 2) again indicating the non-uniformity of these biofilms throughout the duration of the continuous pure culture experiment.

During continuous mode the G+ *C. acetobutylicum *and *E. faecium *biofilms started out slowly and similarly with only small (5 μm high) aggregates of biofilm growth on the electrode. These biofilms did not increase in height like the G- and as time progressed the heights of these biofilms remained low (7-14 μm). By the end of 144 hours the biofilms highest point reached 15 μm, with colony diameters of less than 10 μm. A more detailed description of the pure culture continuous experiments can be seen in Additional file 2. Roughness coefficients for G+ during continuous experiments were higher than those of the batch experiments (Table 2) indicating more non-uniformity during the continuous experiments.

**Figure 4:**
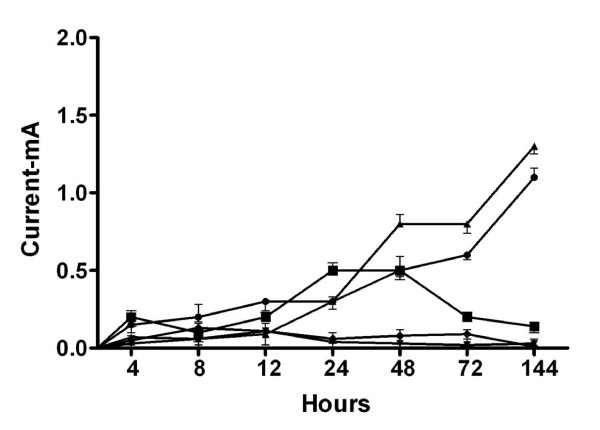
**Pure culture continuous experiment showing Current (mA) vs Time (hours)**. Circle: G. sulfurreducens, Square: P. aeruginosa, Upright triangle: S. oneidensis, Upsidedown triangle: E. faecium and Diamond: C. acetobutylicum

The continuously fed MFCs revealed the G- consistently generating more current than the G+ (Figure 4). *P. aeruginosa *reached its peak in current production (0.5 ± 0.01 mA) between 24-48 hours, however, by 144 hours it had decreased to 0.14 ± 0.01 mA. *G. sulfurreducens *and *S. oneidensis*, on the other hand, both increased current generation later in the experiment while the G+ *E. faecium *and *C. acetobutylicum *maintained a low current throughout.

**Figure 5:**
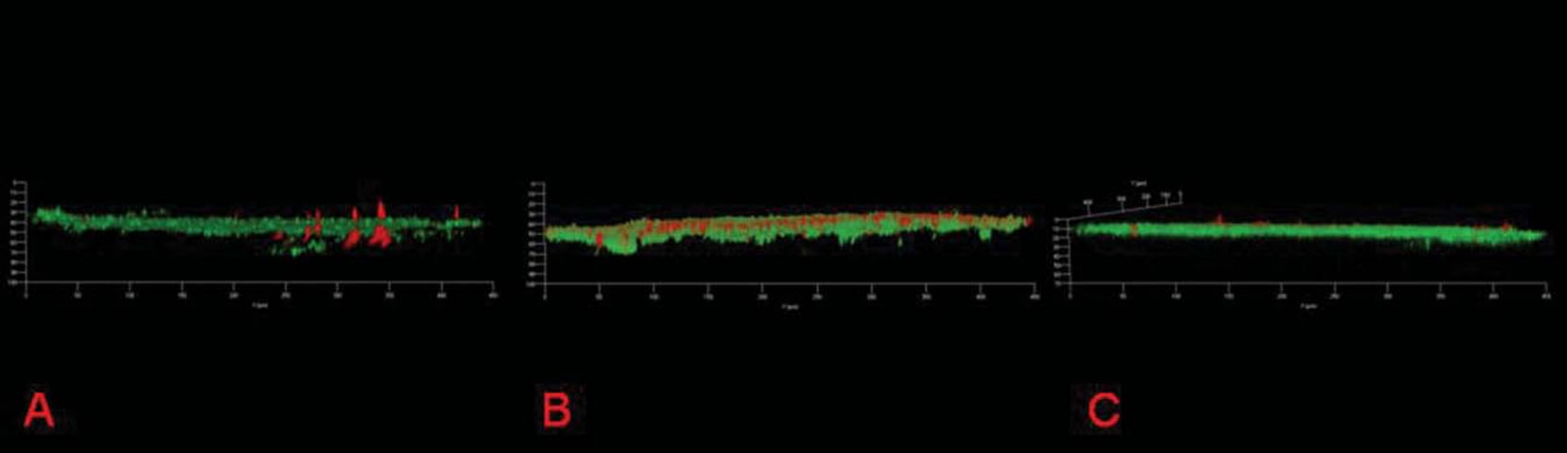
**72 hour FISH confocal microscopy images of Co-cultures A. *P. aeruginosa *(Red) &*E. faecium *(Green) B. *S. oneidensis *(Red) &*E. faecium *(Green) C. *G. sulfurreducens *(Red) &*E. faecium *(Green)**.

During the continuous co-culture experiments, *E. faecium *remained in the close vicinity of the electrode while the G- colonized the top of the biofilm. As time progressed they separated with the G- forming towers and *E. faecium *developed a lawn over the electrode surrounding the G-. Confocal microscopy revealed large towers of *P. aeruginosa *(40 ± 10 μm) surrounded by a lawn of *E. faecium *(Figure 5A). Initially, these towers of *P. aeruginosa *were very sparse and the growth of the two together was patchy although covering more of the electrode than any of the pure cultures. Similarly, *S. oneidensis *and *E. faecium *(Figure 5B) and *G. sulfurreducens *and *E. faecium *co-culture (Figure 5C) biofilms also separated during development with *G. sulfurreducens *and *S. oneidensis *forming smaller towers. A more detailed description of the co-culture experiments is presented in Additional file 3. Roughness coefficients from the co-culture continuous experiments were lower than those of the pure cultures indicating a more uniform and even biofilm (Table 2).

**Figure 6:**
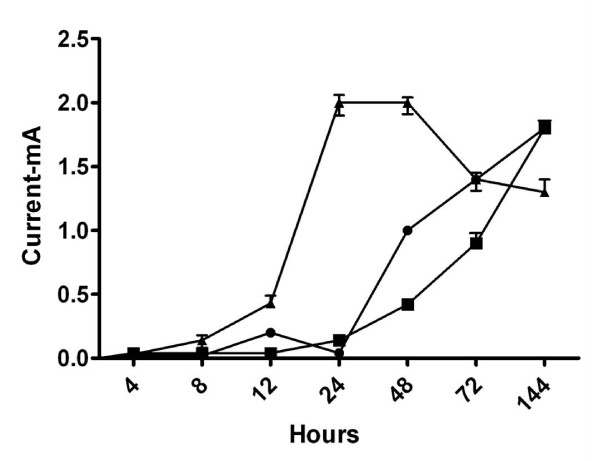
**Current generation (mA) vs Time (Hours) of Co-culture continuous experiment**. Circle: G. sulfurreducens, Square: P. aeruginosa, Upright triangle: S. oneidensis, Upsidedown triangle: E. faecium and Diamond: C. acetobutylicum

Co-culture continuous experiment with *E. faecium *and a G- all produced more current compared to the pure cultures (Figure 6 and Table 1). For example, *S. oneidensis *and *E. faecium *separately generated 1.3 ± 0.05 and 0.1 ± 0.05 mA respectively while together the highest current generated was 2.0 ± 0.06 mA. This co-culture generated more current initially than the *Geobacter *and *Pseudomonas *ones, but levelled off between 24-48 hours after which it began to decrease. This same behaviour was observed across the triplicate experiments. Contrary to *E. faecium*, none of the co-culture experiments with *C. acetobutylicum *showed any difference in performance relative to the pure culture experiments (Table 1).

## Discussion

In this study, we observed quite low current densities relative to a number of dedicated pure culture studies [20]. To accommodate the growth of five different species, we created a joint medium which may have caused suboptimal growth conditions for each culture. However, it eliminated any discrepancies caused by differing constituents within the media when analyzing biofilms. To observe the viability of the anodic biofilms, Live/Dead staining was employed. This stain is an assay for membrane integrity and does not exclusively separate live from dead cells or unequivocally confirms metabolic inactivity [21], nevertheless, it has been successfully used in many studies to indicate viability of the bacteria [22,23]. In this study, this method was thought to be the best option compared to other viability indicators which have to be incubated for a considerable time period or have redox activity by themselves.

### Viability, structure and current of pure culture anode biofilms

During the closed circuit batch experiments viability was maintained in the proximity of the electrode, with slight variations between cultures (Figure 2). This may suggest that the most active part of the biofilm, playing a major role in the EET process, is within 10-20 μm of the electrode. In our set-up, the electron donor was generally provided in excess concentrations. As a result, the decreasing viability away from the anode can rather be attributed to limitations for the electron transfer towards the electrode than substrate limitation. At the current densities observed, it appears unlikely that proton accumulation limited the biofilm performance, as observed previously [24]. During these batch experiments the G- biofilms remained viable while the thinner G+ biofilms rapidly lost viability.

A very insightful study using *G. sulfurreducens *reported no loss of viability as biofilm thickness and current increased [8], while our study revealed a notable increase of the non-viable cells over the duration of the study, with decreasing viability away from the anode. Although this experiment uses the same strain of *G. sulfurreducens *as the Reguera *et al*. (2006) study, the media and fuel cells are not the same which may explain the variations between these two studies.

For all bacteria in the batch experiments, the closed circuit biofilms were thinner than the open circuit biofilms (Table 1). This could be related to the larger thermodynamic gain (e.g. for nitrate E^0' ^= +0.433 V) and availability of the soluble electron acceptors relative to the electrode (anode always below +350 V), which can lead to higher bacterial growth yield [25]. While the biofilm structure of the G- batch experiments was different to the continuous experiments (eg., height and coverage of electrode), the CLSM images indicate that they developed in the typical stages conceptualized in other biofilm studies [17,26], initially as small clusters of biofilm and later as larger towers. The roughness coefficients also differed, suggesting that the biofilms grown in batch mode were more uniform and flatter than those of the continuous experiments, which had higher roughness coefficients. The G+ biofilms were very similar in development during batch and continuous experiments, also in this case the supply of soluble electron acceptors produced thicker biofilms.

The pure cultures of *G. sulfurreducens *and *S. oneidensis *used in this study did not produce as much current as previous studies [8,27]. This may be due to the compromised medium used to grow all five cultures, as well as the suboptimal configuration of the MFC in terms of internal resistance. During all experiments G+ bacteria generated limited current by themselves, while G- bacteria generated much higher currents (Table 1). This was expected as, unlike the G- bacteria, most G+ on their own have limited EET competence [28]. Some are electrochemically active to a certain extent such as a *Clostridium butyricum *strain isolated from a mediatorless MFC [14] and *Enterococcus *sp. [13,18]. In previous work G+ generally require either bacterially produced redox shuttles or humics [29] to generate significant current. One exception to this so far is a thermophilic isolate, *Thermincola *sp. strain JR [30]. In some instances G+ have been seen to dominate populations in mixed culture MFCs [30,31]. Hence, while G+ have some capacity for electron transfer, it is apparent that the G- used here generated much greater current in our MFC conditions. Interestingly, the current generated by *P. aeruginosa *in batch mode was larger than in continuous mode which may be concomitant with the gradual loss of redox shuttles previously implicated in electron transfer by *P. aeruginosa *[10].

*P. aeruginosa *as a pure culture decreased its current production after the 48 hour timepoint (Figure 4) in continuous mode, however, in batch mode it continued to increase current. Potentially, a gradual wash-out of redox shuttles, which can be produced by *P. aeruginosa*, explains the lower performance in continuous mode [32]. A comprehensive, non-MFC based study using PA01 to investigate phenotypic differentiation and seeding dispersal also noted a halt in biofilm height after about 48 hours [33]. During that study microcolonies of 80 μm diameter became differentiated, leaving the microcolony hollow by day 3. Similarly to our current study, by 48 hours PAO1 had formed 20 ± 4 μm thick biofilms, which did not increase throughout the duration of the experiment. Although the aforementioned study used different parameters, the growth and retardation of the PA01 biofilms coincided with the timing of the assumed decreased EET activity in our MFC.

### Co-culture versus pure culture current generation

The three co-cultures (with *E. faecium*) used in this study all generated more current together then when grown as pure cultures. Although this has not yet been investigated at a deeper level, several studies have noted the coexistence between G+ and G- within the MFC environment. For example, the role of a phenazine electron shuttle has been verified in an earlier MFC study where it was observed to increase current generation in co-cultures of *Brevibacillus *sp. and *Enterococcus *sp. with *Pseudomonas *sp. These studies determined that the *G+ *were able to use electron shuttles (mediators) produced by *Pseudomonas *sp [10,28], the combination of both bacteria being the more successful one. Whether other mechanisms such as quorum regulation or the establishment of a syntrophic association is in play is yet to be investigated. In a recent study, Nevin *et al*., [20] described how pure culture biofilms of *G. sulfurreducens *were able to reach current densities of the same order of magnitude as mixed population current densities. In the latter case, the anode surface was minimized in order to ensure that the anode became the limiting factor. It is important to distinguish here that while the electron flux achievable through a defined surface area can indeed be similar for a pure culture and a mixed population, in conditions where the surface area is not limiting (as is the case in our study) mixed populations (or co-cultures) consistently perform better than pure cultures by apparently generating larger reducing power. It is likely that, similar to earlier biofilm studies, metabolic cooperation leads to increased performance [34], further research on this is warranted.

The tower development by the G- organisms in coculture may be an ecological strategy to gain greater access to the carbon source, while maintaining contact with the electrode via a superior electron transfer mechanism. The competition for substrate does not exclude a simultaneous metabolic cooperation for electron transfer. Hansen *et al*., [35] studied the evolution of species within a co-culture and described a symbiotic relationship which in a short duration apparently stabilized species interactions and affected community function. Spatial structure was the key environmental factor provided in our current study as well as in the Hansen study mentioned above. Given suitable conditions to establish a community, the co-cultures used in this study have been allowed to evolve and form their own structure and interactions, which have produced a more productive community.

## Conclusion

This study has shown that biofilms of pure culture G- and G+ remain viable closest to the electrode while becoming non-viable on top or the further away from the electrode. This result was also reiterated by the reverse experiment, where a soluble electron acceptor was offered, with the top of the biofilm remaining viable and the bottom of the biofilm becoming non-viable. The G- cultures developed thicker biofilms, higher towers and produced higher current while the G+ produced thinner biofilms, smaller towers and lower current. Co-culture experiments between *E. faecium *and G- bacteria evidenced a significant increase in current generation when grown together in the MFC, indicating a synergistic or mutualistic relationship between *E. faecium *and G- bacteria within this system which warrants further investigation.

## Methods

### Pure cultures and media

Pure cultures used were *G. sulfurreducens (ATCC 51573)*, *P. aeruginosa *PAO1, *S. oneidensis *MR-1, *C. acetobutylicum *(DSMZ 792) and *E. faecium*. These cultures were all grown in a media containing 0.5 g/L NaCl, 0.1 g/L KCl, 0.2 g/L NH_4_Cl, 0.465 g/L MgSO_4_, 1 ml/L CaCl_2_, 2 g/L NaHCO_3_, 6 g/L Na_2_HPO_4_, 3 g/L KH_2_PO_4_, 0.05 g/L yeast extract, 10 ml/L vitamin solution (Sigma-Aldrich Pty. Ltd., Castle Hill, Australia), 10 ml/L of trace element solution [36], 20 mM of sodium acetate (Sigma) and 20 mM lactate (Sigma). For the experiments in which the anode was not conveying any current (open circuit), 20 mM nitrate and 40 mM fumarate were supplied as electron acceptors. The catholyte was a 100 mM solution of potassium ferricyanide (K_3 _[Fe (CN)_6_]. Cultures were pre-grown to mid exponential phase (determined by OD 600 nm measurement) in the same media using soluble electron acceptors (nitrate and fumarate). They were then centrifuged (for 10 mins @ 5,000 g) to obtain a pellet which was washed in the above media without electron acceptors and again centrifuged (10 mins @ 5,000 g). The supernatant was then decanted, replaced with fresh media and 1 ml of this culture was used to inoculate the MFC. For the co-culture experiments the method was the same as the pure culture with 500 μl of each culture being added to the reactor.

### Microbial fuel cells and electrochemical measurements

Plate type reactors were constructed as described in Aelterman et al., [31] with an anode volume of 336 cm^3^. The modification to this reactor design as used in this study was the addition of removable side panels for sample collection and only two cathode and anode compartments. A cation exchange membrane (Ultrex CMI-7000, Membranes International, USA) was placed between the anode and cathode compartments and rubber seals were used to securely seal the compartments. Granular graphite with diameter ranging between 2 and 6 mm (El Carb 100, Graphite Sales, Inc., USA) was used in the cathode compartment as an electrode with a graphite rod through each compartment used for external connection. The granules were initially left overnight in 1 M HCl, washed with deionized water, left overnight again in 1 M NaOH and then washed several times in deionized water. The total empty volume of the cathode compartment was 336 cm^3 ^and approximately 182 cm^3 ^when the granules were added. The anode electrode had the same type of graphite rod, which connected to twelve 2 cm × 1 cm × 1 cm graphite blocks, one 10 cm × 2 cm × 1 cm and one 10 cm × 1 cm × 1 cm graphite blocks to make up the total electrode surface area of 72 cm^2 ^used for sampling. These blocks were initially lightly smoothed with fine grade wet/dry sandpaper, washed and autoclaved. The electrode arrangement is shown in Figure 1. The voltage over the MFCs was monitored using an Agilent 34970A data acquisition unit. A full channel scan was performed every 30 s and data was stored. External resistance was 100, all calculations were performed according to Rabaey et al., [37] and Logan et al., [38].

### MFC Reactor operation

Initially, a series of MFC batch experiments was performed in triplicate for each bacterial strain in the presence (closed circuit) and absence (open circuit) of external current. These batch reactors used recirculated media and were operated for three days. This time point was chosen as during optimization of the experiments, the highest current peak was achieved during this time. MFCs were sterilized by flushing with household bleach (50% with MiliQ water) over night and then recirculated with sterile MilliQ for two days, to ensure all residual bleach was removed, followed by UV irradiation. Anodes and cathodes of the reactors were flushed prior to the experiment with nitrogen gas to create anaerobic conditions. Then the anode was filled with anaerobic autoclaved media, with no soluble electron acceptor for the closed circuit experiments, while the cathode was filled with anaerobic catholyte. The anodes were then inoculated with the pure cultures and anodes and cathodes were connected over a resistance of 100 Ω. After three days the MFCs were disconnected and blocks were taken from the removable side panel under anaerobic conditions. For the open circuit experiments the same reactor set-up was used except the anodes were not connected to the cathode and the soluble electron acceptors fumarate and nitrate were added at final concentrations of 20 mM. The open circuit experiments were run for three days at which time blocks were again collected.

Continuous experiments were run for 144 hours (in triplicate) with blocks taken for sampling at 0, 4, 8 12, 24, 72 and 144 hours under anaerobic conditions. These time points were chosen based on current literature [39,40] and possible developmental changes within the biofilm as seen during optimization of these experiments. These experiments were conducted in duplicate under the same conditions as the closed circuit batch experiments using the same media but continuously fed at a recirculated flow rate of 0.8 L/day. Inoculum for the continuous MFCs was the same as those for the batch experiments, with the addition that for the co-culture experiments the mixtures of the pure cultures were used.

### Fluorescent *in-situ *Hybridisation (FISH) and viability staining

During the continuous experiments one anodic graphite block from each reactor was regularly collected for FISH analysis. When blocks were initially taken from the reactors, they were washed with basic media that did not include electron donor or acceptor to remove any particulates that may auto fluoresce. FISH sample fixation, hybridization and washing was performed as described previously [41]. Blocks were visualized using the CLSM (Zeiss LSM510) and a 20 × objective to obtain an overall view of the biofilm. Probes used were Pae997 (Cy3-35% Formamide (F)) (*P. aeruginosa*) (G-) (5'-TCT GGA AAG TTC TCA GCA-3') [42], GEO-2 (Cy3-35% F) (*G. sulfurreducens*) (G-) (5'-GAA GAC AGG AGG CCC GAA A-3') with helper probe HGEO-2 (5'-GTC CCC CCC TTT TCC CGC AAG A-3') [43], SPN3 (Cy3-35% F) (*S. oneidensis*) (G-) (5'-CCG GTC CTT CTT CTG TAG GTA ACG TCA CAG-3') [44], EFA-1 (FITC-35% F) (*E. faecium*) (G+) (5'-TGA TTT GAA AGG CGC TTT CGG GTG TCG CTG ATG GAT GGA C-3') [45] and LGC354B (FITC-35% F) (*C. acetobutylicum*) (G+) (5'-CGG AAG ATT CCC TAC TGC-3') [46].

The BacLight™ Bacterial Viability Kit (Invitrogen, Mount Waverley, Australia) was used on all pure cultures for batch and continuous studies. Again, one block from each reactor was collected at each time point for Live/Dead analysis and washed with media to remove any particulates. The stain was placed immediately on top of the graphite blocks when removed from the reactor and then washed with the same media after 10 minutes to remove excess stain. These were visualised using the Zeiss LSM510 Confocal Laser Scanning Microscope (CLSM) with a 20 × objective.

CLSM visualization was used to determine biofilm height and to create 3D biofilm images using 20 × objective to obtain an overall view of the biofilm. These images were then used to determine percentage viability and biofilm coverage using pixel counting with the aid of Adobe Photoshop. Three random representative images were taken from each block used for FISH and Live/Dead staining. The 3D images were created from 1 μm z-stacks slices of varying heights (depending on the height of the biofilm) and were constructed using Zeiss 3D imaging software.

### SEM analysis

During co-culture experiments blocks (2 mm wide) were removed from the reactors at 72 and 144 hour time points and fixed immediately for SEM analysis. SEM fixation involves the use of 3 solutions. Solution 1 contains 0.043 g lysine (L-lysine free base Sigma L-5501) dissolved in 2 ml of 0.1 M cacodylate buffer. Solution 2 contains 0.4 ml 25% glutaraldehyde, 1.0 ml 0.2 M cacodylate buffer and 0.6 ml distilled water. Solutions 1 and 2 were mixed together thoroughly immediately before use. Samples were left in this for 10 minutes then transferred to solution 3 which is 2.5% glutaraldehyde in 0.1 M cacodylate buffer for further sample processing as described in Jacques & Graham [47]. Samples for SEM were visualized using JEOL JSM- 6400F microscope (10 kV, 3000 V) and EIKO IB-5 sputter coater using platinum.

### COMSTAT analysis of biofilms

Z-stacks generated using the CLSM were further analysed using COMSTAT to determine roughness coefficient and mean biofilm thickness. Through COMSTAT a fixed threshold was applied to the images to provide a 0 or 1 value to image pixels. One represents areas containing biomass while 0 is considered as background [48]. The thickness function is the maximum thickness over a given location which does not take into account any pores or voids within the biofilm. The thickness distribution is then used to calculate the biofilm roughness and mean biofilm thickness. Roughness coefficient provides an indication of how the thickness of the biofilm varies and also provides an indication of biofilm heterogeneity [48].

## Authors' contributions

SR completed all the reactor and biofilm experiments and analysis and wrote the manuscript, KR contributed with the design of the study, designed the reactors and technical support throughout; PD performed all the SEM; JK, PB were involved in editing and revising the manuscript critically in preparation for submission. All authors read and approved the final manuscript.

## Supplementary Material

Additional file 1 **CLSM top view cropped image of *S. oneidensis *biofilm **(Figure 2) **(63×) providing a close-up of the nonviable cells using Live/Dead (Baclight) stain**. Additional File 1 is a more detailed confocal image of the *S. oneidensis *biofilm. Its purpose is to show the difference between live and dead cells after using the Live/Dead stain. Click here for file

Additional file 2 **Observations of Pure culture continuous time course biofilm study**. A table describing the development of the pure culture biofilms during the continuous experiment. Click here for file

Additional file 3 **Observations of Co-culture continuous time course biofilm study**. A table describing the development of the co-culture biofilms during the continuous experiment. Click here for file
