# Supplementary figures and images for: Initial development and structure of biofilms on microbial fuel cell anodes
Source: BMC Microbiol. 2010 Apr 1;10:98. doi: 10.1186/1471-2180-10-98 (PMC2858741; doi:10.1186/1471-2180-10-98)

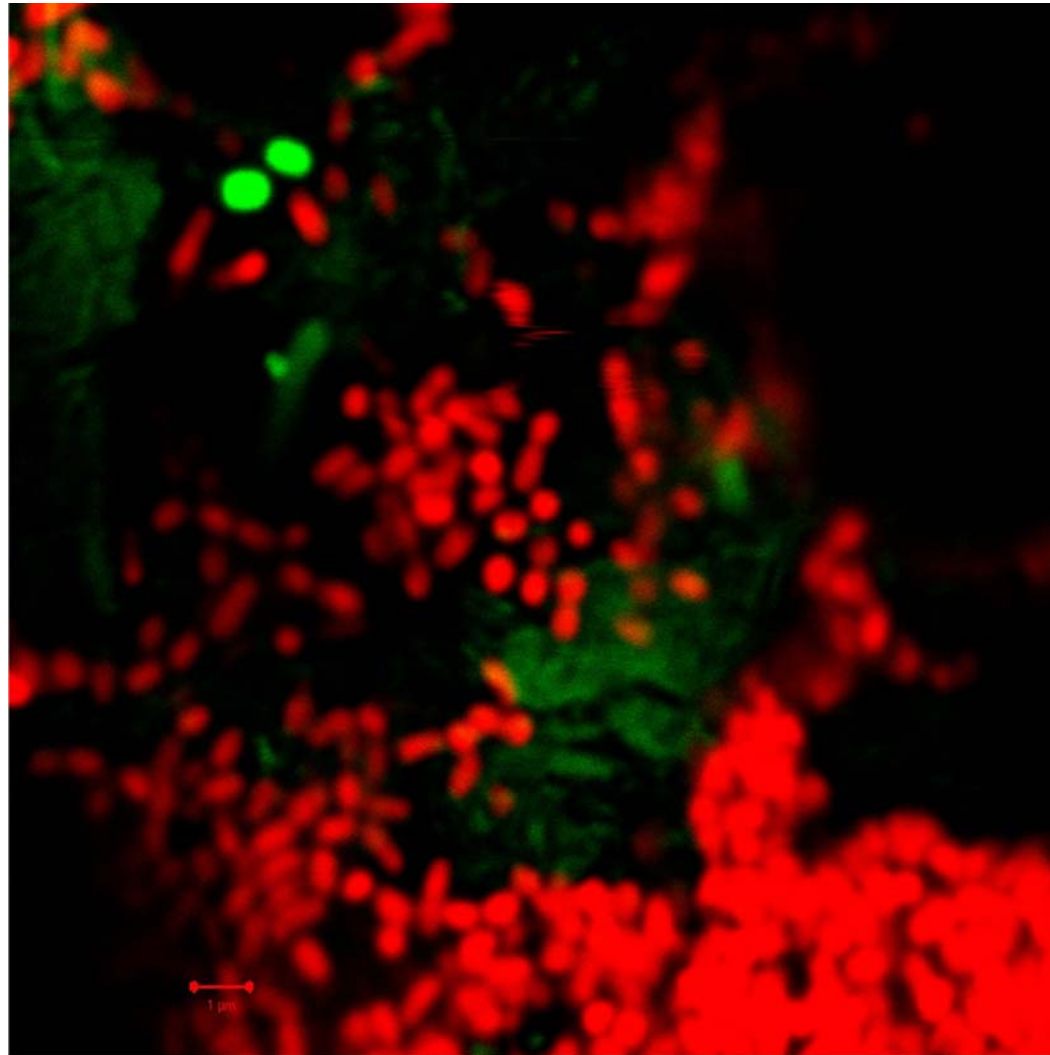

Supplement: Additional file 1 — CLSM top view cropped image of S. oneidensis biofilm (Figure 2) (63×) providing a close-up of the nonviable cells using Live/Dead (Baclight) stain. Additional File 1 is a more detailed confocal image of the S. oneidensis biofilm. Its purpose is to show the difference between live and dead cells after using the Live/Dead stain. [file 1471-2180-10-98-S1.PDF]
