# Supplementary material for: Initial development and structure of biofilms on microbial fuel cell anodes
Source: BMC Microbiol. 2010 Apr 1;10:98. doi: 10.1186/1471-2180-10-98 (PMC2858741; doi:10.1186/1471-2180-10-98)
Supplement: Additional file 2 — Observations of Pure culture continuous time course biofilm study. A table describing the development of the pure culture biofilms during the continuous experiment. [file 1471-2180-10-98-S2.PDF]

Additional File 2. Observations of Pure culture continuous time course biofilm study

Organism	Hours of biofilm growth	Coverage of electrode-%	Height of Biofilm (µm) COMSTAT	Colony type (eg. Micro up to 10µm, Macro above 10µm diameter)	Biofilm features	Tower size-maximum (µm) and average number observed over three separate images
G.sulfurreducens	4	5	5±2	Micro	Small cluster in one corner	10-(5)
P.aeruginosa	4	50	15±5	Micro (5%) and Macro (20±15µm)	Sporadic, non-uniform coverage of electrode surface	20-(6)
S.oneidensis	4	30	9±5	Micro	Little coverage on surface, more non-viable than others	14-(3)
E.faecium	4	10	0-5	Micro	Small colonies	10-(3)
C. acetobutylicum	4	10	0-5	Micro	Small colonies	5-(3)
G.sulfurreducens	8	80	15 ±5	Macro/Biofilm formed	Uneven lawn coverage	20-(3)
P.aeruginosa	8	50	15±5	Micro (5%) and Macro (40±20µm)	Similar Coverage as 4hours	25-(4)
S.oneidensis	8	40	20±5	Micro (50%) and Macro (40±20µm)	Little coverage on surface, still more non-viable than others	25-(3)
E.faecium	8	20	4±4	Micro (20%) and Macro (20±10µm)	Small colonies	8-(3)
C. acetobutylicum	8	40	7±5	Micro (30%) and macro (20±5µm)	Little coverage of electrode	8-(3)

**Organism**	**Hours of biofilm growth**	**Coverage of electrode-%**	**Height of Biofilm (µm) COMSTAT**	**Colony type (eg. Micro up to 10µm, Macro above 10µm diameter)**	**Biofilm features**	**Tower size-maximum (µm) and average number observed over three separate images**
G.sulfurreducens	12	80	25 ±5	Macro/Biofilm formed	Mostly covered, a few patchy areas due to channel formation	35-(1)
P.aeruginosa	12	90	20±10	Macro/Biofilm formed	Coverage of electrode increased, small non-viable towers	30-(3)
S.oneidensis	12	50	20±9	Micro (20%) and Macro (100±20µm)	More coverage on surface although very sporadic	30-(4)
E.faecium	12	40	5±4	Micro (5%) and Macro (60±20µm)	Little coverage of electrode	8-(5)
C. acetobutylicum	12	50	7±3	Macro (40±20µm)	Little coverage of electrode	15-(3)
G.sulfurreducens	24	75	25 ±7	Macro/Biofilm formed	Obvious channels and towers	40-(1)
P.aeruginosa	24	95	20±10	Macro/Biofilm formed	Same as above	30-(5)
S.oneidensis	24	60	30±5	Macro/biofilm formed	More coverage on surface and channel formation	35-(4)
E.faecium	24	40	11±3	Macro (100±50µm)	Larger colonies but still little coverage	No obvious towers observed
C. acetobutylicum	24	80	11±5	Macro (120±50µm)	Uniform flat biofilm, higher structures not observed at this timepoint	No obvious towers observed

Organism	Hours of biofilm growth	Coverage of electrode-%	Height of Biofilm (µm) COMSTAT	Colony type (eg. Micro up to 10µm, Macro above 10µm diameter)	Biofilm features	Tower size-maximum (µm) and average number observed over three separate images
G.sulfurreducens	48	75	25±7	Macro/Biofilm formed	Similar morphology as previous	40-(1)
P.aeruginosa	48	65	20±10	Macro/Biofilm formed	Channel formation and loss of biomass	30-(5)
S.oneidensis	48	70	20±10	Macro/biofilm formed	Channels but still very sporadic biofilm	40-(2)
E.faecium	48	60	9±3	Macro/Biofilm formed	Uniform flat biofilm, higher structures not observed at this timepoint	No obvious towers observed
C. acetobutylicum	48	70	9±6	Macro/Biofilm formed	Uniform flat biofilm, higher structures not observed at this timepoint	No obvious towers observed
G.sulfurreducens	72	60	28±10	Macro/Biofilm formed	Increase in non-viable cells, obvious biomass detachment	50-(1)
P.aeruginosa	72	70	20±10	Macro/biofilm formed	More defined channels and patterns observed	35-(4)
S.oneidensis	72	60	20±10	Macro/Biofilm formed	Channels maintained, very patchy	45-(2)
E.faecium	72	60	9±3	Macro/Biofilm formed	Flat, messy lawn, no recognisable strucutres	No obvious towers observed
C. acetobutylicum	72	60	9±4	Large flat Macro (100±20µm)	Flat, messy lawn, no recognisable strucutres	No obvious towers observed

**Organism**	**Hours of biofilm growth**	**Coverage of electrode-%**	**Height of Biofilm (µm) COMSTAT**	**Colony type (eg. Micro up to 10µm, Macro above 10µm diameter)**	**Biofilm features**	**Tower size-maximum (µm) and average number observed over three separate images**
G.sulfurreducens	144	60	20±8	Some micro (5%) and Macro (150±150µm diameter)	Very patchy with large towers	45-(2)
P.aeruginosa	144	95	10±5	Macro/Biofilm formed	Lawn coverage of electrode	15-(3)
S.oneidensis	144	60	25±10	Macro/Bioiflm formed	Channels maintained, very patchy	48-(2)
E.faecium	144	50	9±5	Macro/Biofilm formed	Flat, messy lawn, no recognisable strucutres	No obvious towers observed
C. acetobutylicum	144	60	9±4	Macro/Biofilm formed	Flat, messy lawn, no recognisable strucutres	No obvious towers observed
