# Supplementary material for: Initial development and structure of biofilms on microbial fuel cell anodes
Source: BMC Microbiol. 2010 Apr 1;10:98. doi: 10.1186/1471-2180-10-98 (PMC2858741; doi:10.1186/1471-2180-10-98)
Supplement: Additional file 3 — Observations of Co-culture continuous time course biofilm study. A table describing the development of the co-culture biofilms during the continuous experiment. [file 1471-2180-10-98-S3.PDF]

**Additional File 3.** Observations of Co-culture continuous time course biofilm study

| HOURS | *P.aeruginosa*(PA) and *E.faecium* (EF) Co-culture | PA/EF co-culture biofilm height (µm) COMSTAT | *S.oneidensis* (SO) and *E.faecium* (EF) Co-culture | SO/EF co-culture biofilm height (µm) COMSTAT | *G.sulfurreducens* (GS) and *E.faecium* (EF) Co-culture | GS/EF co-culture biofilm height (µm) COMSTAT |
|---|---|---|---|---|---|---|
| **4** | -PA complete coverage on top of biofilm<br>-Couple of PA towers 25-30µm high<br>-EF covering electrode underneath PA<br>-Biofilm PA/EF- 2-5/10-15µm high<br>-70% coverage of electrode | 15±5/0-5 | -SO complete coverage on top of biofilm<br>-a number of small SO towers 10-12µm high<br>-EF covering electrode underneath SO<br>-Biofilm SO/EF- 2-5/10-15µm high<br>-40% coverage of electrode | 9±5/0-5 | -GS on top of biofilm<br>-No towers<br>-EF covering electrode underneath GS<br>-Biofilm GS/EF-2-5/10-15µm high<br>-70% coverage of electrode | 5±2/0-5 |
| **8** | -PA complete coverage on top of biofilm<br>-Couple of PA towers 35-40µm high<br>-EF covering electrode underneath PA<br>-Biofilm PA/EF- 2-5/15-25µm high<br>-70% coverage of electrode | 15±5/4±2 | -SO complete coverage on top of biofilm<br>-Larger SO towers 25-30µm high<br>-EF covering electrode underneath SO<br>-Biofilm SO/EF- 2-5/20-25µm high<br>-70% coverage of electrode | 15±5/4±4 | -GS on top of biofilm<br>-No towers<br>-EF covering electrode underneath GS<br>-Biofilm GS/EF-2-5/10-15µm high<br>-80% coverage of electrode | 15 ±5/4±4 |
| **12** | -Very small amount of PA on top of biofilm<br>-Couple of PA towers 25-30µm high<br>-EF covering electrode<br>-Biofilm PA/EF-0-5/10-25µm high<br>-90% coverage of electrode | 20±5/5±4 | -Little SO on top of biofilm<br>-a number of small SO towers 10-15µm high<br>-EF covering electrode underneath SO<br>-Biofilm SO/EF- 0-5/8-12µm high<br>-90% coverage of electrode | 20±9/5±4 | -GS on top of biofilm<br>- a number of small GS towers attached to electrode 8-10µm high<br>-Less EF covering electrode underneath GS,<br>-Biofilm GS/EF-2-5/10-15µm high<br>-80% coverage of electrode | 25 ±5/5±4 |

| **HOURS** | ***P.aeruginosa* (PA) and *E.faecium* (EF) Co-culture** | **PA/EF co- culture biofilm height (µm) COMSTAT** | ***S.oneidensis* (SO) and *E.faecium* (EF) Co-culture** | **SO/EF co-culture biofilm height (µm) COMSTAT** | ***G.sulfurreducens* (GS) and *E.faecium* (EF) Co-culture** | **GS/EF co-culture biofilm height (µm) COMSTAT** |
|---|---|---|---|---|---|---|
| **24** | -Little PA on top of biofilm<br>-Number of PA towers 40-50µm high<br>-EF covering electrode<br>-Biofilm PA/EF-0-5/10-20µm high<br>-90% coverage of electrode | 20±5/18±3 | -Little SO on top of biofilm<br>-number of small 10-20µm high SO towers<br>attached to electrode<br>-EF still covering electrode although patchier than previous time points<br>-Biofilm SO/EF-0-2/10-15µm high<br>-70% coverage of electrode | 30±5/18±3 | -More GS on top of biofilm<br>-GS Towers have formed 25-30µm high<br>-EF still covering electrode although patchier than previous time points-A lot thinner than the other co-cultures<br>-Biofilm GS/EF-5-8/10-15µm high<br>-70% coverage of electrode | 30 ±7/18±3 |
| **48** | -Little PA on top of biofilm<br>-A number of PA of towers 35-40µm high<br>-EF covering electrode<br>-Biofilm PA/EF- 0-3/15-20µm high<br>-80% coverage of electrode | 20±5/15±7 | -Little SO on top of biofilm<br>-a number of SO towers 20-25µm high<br>-EF covering electrode<br>-Biofilm SO/EF-0-2/10-25µm high<br>-70% coverage of electrode | 30±4/15±7 | -Some GS on top of biofilm<br>-A number of GS towers 20-30µm high<br>-EF covering electrode underneath GS<br>-Biofilm GS/EF-2-5/20-25µm high<br>-60% coverage of electrode | 25±7/15±7 |

| **HOURS** | ***P.aeruginosa* (PA) and *E.faecium* (EF) Co-culture** | **PA/EF co- culture biofilm height (µm) COMSTAT** | ***S.oneidensis* (SO) and *E.faecium* (EF) Co-culture** | **SO/EF co-culture biofilm height (µm) COMSTAT** | ***G.sulfurreducens* (GS) and *E.faecium* (EF) Co-culture** | **GS/EF co-culture biofilm height (µm) COMSTAT** |
|---|---|---|---|---|---|---|
| **72** | -No PA on top of biofilm<br>-A number of PA towers 25-30µm high<br>-Less EF covering electrode<br>-Biofilm PA/EF – 0/20-30µm high<br>-70% coverage of electrode | 13±8/16±3 | -Some SO still on top of biofilm<br>-a number of SO towers 10-20µm high<br>-EF covering electrode underneath SO<br>-Biofilm SO/EF- 0-2/10-15µm high<br>-70% coverage of electrode | 20±8/16±3 | -Some GS still on top of biofilm<br>-Some large GS towers 25µm high<br>-EF still covering electrode underneath GS<br>-Biofilm GS/EF-2-5/10-20µm high<br>-90% coverage of electrode | 20±7/16±3 |
| **144** | -No PA on top of biofilm<br>-Large PA towers 40-50µm high<br>-EF still covering electrode<br>-Biofilm PA/EF -0/20-30µm high<br>-80% coverage of electrode | 10±5/10±5 | -Small amount of SO still on top of biofilm<br>-a number of small SO towers 10-25µm high<br>-EF covering electrode although patchier<br>-Biofilm SO/EF-0-2/10-20µm high<br>-70% coverage of electrode | 25±10/10±5 | -No GS on top of biofilm<br>-Some large GS towers 25-30µm high, a number of small GS towers 10-15µm high<br>-EF still covering electrode<br>-Biofilm GS/EF-0/10-30µm high<br>-80% coverage of electrode | 20±6/10±5 |
